# Drop-the-*p*: Bayesian CFA of the Multidimensional Scale of Perceived Social Support in Australia

**DOI:** 10.3389/fpsyg.2021.542257

**Published:** 2021-02-26

**Authors:** Pedro Henrique Ribeiro Santiago, Adrian Quintero, Dandara Haag, Rachel Roberts, Lisa Smithers, Lisa Jamieson

**Affiliations:** ^1^Australia Research Centre for Population Oral Health, Adelaide Dental School, The University of Adelaide, Adelaide, SA, Australia; ^2^School of Public Health, The University of Adelaide, Adelaide, SA, Australia; ^3^Icfes – Colombian Institute for Educational Evaluation, Bogotá, Colombia; ^4^School of Psychology, The University of Adelaide, Adelaide, SA, Australia

**Keywords:** Bayesian confirmatory factor analysis, Bayesian inference, social support, Australia, psychometrics

## Abstract

**Aim:**

We aimed to investigate whether the 12-item Multidimensional Scale of Perceived Social Support (MSPSS) constitutes a valid and reliable measure of social support for the general adult Australian population.

**Methods:**

Data were from Australia’s National Survey of Adult Oral Health 2004–2006 and included 3899 participants aged 18 years old and over. The psychometric properties were evaluated with Bayesian confirmatory factor analysis. One-, two-, and three-factor (Significant Other, Family and Friends) structures were tested. Model fit was assessed with the posterior predictive *p*-value (PPP_χ2_), Bayesian root mean square error of approximation (BRMSEA), and Bayesian comparative fit index (BCFI). Dimensionality was tested by comparing competing factorial structures with the Bayes factor (BF). Reliability was evaluated with the Bayesian Ω_*H*_. Convergent validity was investigated with the Perceived Stress Scale (PSS) and discriminant validity with the Perceived Dental Control scale (PDC-3).

**Results:**

The theoretical three-factor model (Significant Other, Family, and Friends) provided a good fit to the data [PPP_χ2_ < 0.001, BRMSEA = 0.089-95% credible interval (CrI) (0.088, 0.089); BCFI = 0.963-95% CrI (0.963, 0.964)]. The BF provided decisive support for the three-factor structure in relation to the other structures. The SO [BΩ_*H*_ = 0.95 - 95% CrI (0.90, 0.99)], FA (BΩ_*H*_ = 0.92 - 95% CrI (0.87, 0.97), and FR (BΩ_*H*_ = 0.92 - 95% CrI (0.88, 0.97)] subscales displayed excellent reliability. The MSPSS displayed initial evidence of convergent and discriminant validity.

**Conclusion:**

The MSPSS demonstrated good psychometric properties and excellent reliability in a large Australian sample. This instrument can be applied in national surveys and provide evidence of the role of social support in the Australian population.

## Introduction

Social support is a key social determinant of health (Wilkinson and Marmot, 2003). It works as a buffer of life adversities through multiple mechanisms, including supportive actions of others and the belief that support is available (Cohen et al., 2000). Substantial evidence has shown that increased social support is associated with a reduction in the effect of stressful events, higher self-regulation, and better physical and psychological health outcomes (Wilkinson and Marmot, 2003). Since social support is a complex and multidimensional construct (Lakey and Cohen, 2000), a number of instruments have been developed to measure different aspects of social support (van Dam et al., 2005). For instance, instruments were created to evaluate social support functions (e.g., emotional, tangible, positive interaction, companionship) (Sherbourne and Stewart, 1991), sources of social support (e.g., signification other, family, friends) (Zimet et al., 1988), received (e.g., a friend loaned money) (Barrera et al., 1981) or perceived (e.g., there are people I can depend on) social support (Cutrona and Russell, 1987), social support availability (Cohen and Hoberman, 1983) or adequacy, among others (Gottlieb and Bergen, 2010). Thus, although there is no single instrument that covers every aspect of social support, a review indicated five social support measures with strong psychometric properties (López and Cooper, 2011).

One of these five measures is the Multidimensional Scale of Perceived Social Support (MSPSS), a 12-item instrument originally developed by Zimet et al. (1988) to evaluate perception of social support adequacy from three different sources (significant other, family, and friends). Since its development, the MSPSS has been widely adopted, being translated to more than 20 languages, due to several reasons (Dambi et al., 2018). First, it measures the perception of social support (e.g., quality of relationships), in line with empirical findings that the quality of support is a better predictor of psychological status than support objectively measured (Wu et al., 2011). Second, the MSPSS evaluates distinct sources of social support, including “family,” “friends,” and “significant other,” which might elucidate the different mechanisms through which social support operates to improve health and other psychosocial outcomes (Bruwer et al., 2008). Taken together, the abovementioned characteristics of the MSPSS help inform interventions focused on distinct aspects of social support that are relevant to specific outcomes in the population.

### MSPSS Psychometric Properties

In the original validation of the MSPSS, principal component analysis (PCA) indicated a three-factor structure, the Significant Other (SO), Family (FA), and Friends (FR) subscales, which displayed good internal consistency (α_*SO*_ = 0.91, α_*FA*_ = 0.87, α_*FR*_ = 0.85) and adequate test–retest reliability (α_*SO*_ = 0.72, α_*FA*_ = 0.85, α_*FR*_ = 0.75). Soon after the initial study, two additional validations were conducted by the instrument developers Zimet, Powell (Zimet et al., 1990) and Dahlem, Zimet (Dahlem et al., 1991) in independent samples, reporting again the three-factor structure and good reliability.

Over the decades, the psychometric properties of the MSPSS have been evaluated worldwide, including countries such as Pakistan (Akhtar et al., 2010), China (Chou, 2000), Uganda (Nakigudde et al., 2009), Mexico (Edwards, 2004), Turkey (Başol, 2008), Thailand (Wongpakaran et al., 2011), Nepal (Tonsing et al., 2012), South Africa (Bruwer et al., 2008), Malaysia (Ng et al., 2010), Iran (Bagherian-Sararoudi et al., 2013), Sweden (Ekbäck et al., 2013), Russia (Pushkarev et al., 2018), and others (Denis et al., 2015; Theofilou, 2015). Despite being investigated in multiple countries, a recent systematic review by Dambi, Corten (Dambi et al., 2018) indicated several limitations of previous MSPSS validations and cross-cultural adaptations. Previous MSPSS validations were conducted in small- or medium-sized samples of restricted populations, mostly clinical and university convenience samples. For example, the MSPSS properties were previously investigated in 275 undergraduate students (Zimet et al., 1988), 325 pregnant women (Akhtar et al., 2010), 475 high school students (Chou, 2000), 433 school administrators (Başol, 2008), 310 medical students and 152 psychiatric patients (Wongpakaran et al., 2011), 176 myocardial infarction (MI) patients (Bagherian-Sararoudi et al., 2013), 127 women with hirsutism and 154 nursing students (Ekbäck et al., 2013), among others.

Another point discussed by Dambi, Corten (Dambi et al., 2018) was the MSPSS factorial structure. The majority of previous studies reported the three-factor structure with few notable exceptions: Lai, Hamid (Lai et al., 1996) and Chou (2000) reported a two-factor structure, which combines the SO with the FR subscale, in Chinese samples. The reason for combining the subscales was that all SO items use the term “special person” (e.g., “There is a special person who is around when I am in need”), and respondents believed that a “special person” could be referring to a friend. The conceptual overlap happened because the “person we call ‘special’ might differ according to culture” (Başol, 2008) and the Chinese respondents did not distinguish between the two terms. For this reason, Eker, Arkar (Eker et al., 2001) suggested that an explanation for the “special person” term (“a girlfriend/boyfriend, fiancé, relative, neighbour, or a doctor”) should be added to the items to provide clarification. Additionally, one-factor MSPSS structures were also reported in certain “collectivistic” societies, such as rural areas in Pakistan, in which the “sense of communal living dilutes the differences between family members, friends and significant others” (Akhtar et al., 2010).

Moreover, in the majority of previous MSPSS validations, the method employed to investigate the dimensionality was exploratory factor analysis (EFA). While the replication of the three-factor structure (Significant Other, Family, and Friends) by EFA in multiple independent studies provides support for the original MSPSS dimensionality, Dambi, Corten (Dambi et al., 2018) pointed out limitations on how EFA was used. For instance, some studies employed EFA with orthogonal rotation (instead of oblique rotation) (Nakigudde et al., 2009; Ekbäck et al., 2013, 2014), which assumes uncorrelated factors. The problem is that moderate correlations are expected between receiving support from a Significant Other, Family, and/or Friends, and these three factors were shown to be correlated in previous MSPSS literature (Dambi et al., 2018).

Dambi, Corten (Dambi et al., 2018) also discussed limitations in the investigation of model fit. Fit indices traditionally employed in factor models, such as the root mean square error of approximation (RMSEA) (Steiger, 1980), have been evaluated in the context of EFA (e.g., factor retention) (Preacher et al., 2013; Barendse et al., 2015) and are available for EFA in modern software, such as R package *psych* (Revelle, 2017). Despite its availability, the majority of MSPSS validations that employed EFA did not report fit indices. The problem is that, for the studies that reported the three-factor structure, it is not possible to know “the degree to which the data/translation fits into the original factor model” (Dambi et al., 2018). That is, it is not possible to know whether the three-factor structure was actually a good fit for the data. In case of poor fit, alternative MSPSS factorial structure, such as the two-factor structure (Friends and Family), or model respecifications, such as the inclusion of correlated uniqueness (Brown, 2014), would have to be considered.

For these reasons, Dambi, Corten (Dambi et al., 2018) argued that, for the investigation of the MSPSS dimensionality, confirmatory factor analysis (CFA) should be preferred since it enables the evaluation of *a priori* MSPSS theoretical structures and “given that the MSPSS can yield one-, two- or three-factors, all the three models should be tested using CFA before a decision on the degree of fit can be made.” However, a minority of previous MSPSS validations performed CFA, and of these, only three studies adequately described fit indices. The authors concluded that “provision of multiple goodness-of-fit indices for all the three (MSPSS) models should be a ‘standard’ reporting practise as it provides the potential readership with all the essential information for them to critique the methodological quality and subsequent conclusions in keeping with the evidence supplied” (Dambi et al., 2018).

In summary, while the replication of the three-factor model in multiple independent studies indicates support for the original MSPSS factorial structure, a recent review recommended that future MSPSS validations should provide more robust evidence regarding the fit of the original three-factor model and its comparison to alternative MSPSS factorial structures (e.g., two-factor model, one-factor model).

### The Present Research

Considering the shortcoming of previous MSPSS validations, there are three gaps in the literature that this study intends to address: first, the MSPSS validation studies were conducted in small- or medium-sized convenience samples of restricted populations. To the best of our knowledge, there are no studies that investigated the MSPSS validity for a general population using a large sample. While convenience samples do not necessarily lead to biased estimates (i.e., biased factor loadings) (Rothman et al., 2013), generalizability to a national population is unclear (Jager et al., 2017). For instance, validation studies of other instruments showed “slight differences in the strength of associations with other constructs in convenience and representative samples,” warranting further investigation (Leckelt et al., 2018). In Australia, one study validated an instrument (to measure perceived stress) in a restricted Indigenous subpopulation reporting good psychometric properties (Santiago et al., 2019) that have not been replicated at a national level (Santiago et al., 2020). In conclusion, while it is possible that psychometric properties of an instrument generalize from restricted subgroups to the national population, independent validation in national samples still seems to be required. In the case of the MSPSS, the examination in a general population can inform, for example, whether this instrument is suitable for application in large, population-level social support interventions.

Second, despite the MSPSS being previously used in epidemiological research in Australia (O’Dea and Campbell, 2011; Schuurmans-Stekhoven, 2017), there were no psychometric studies that evaluated its construct validity specifically for the Australian population. One important characteristic of the MSPSS compared to other social support instruments is that it provides information about distinct sources of social support, such as significant other, family, and friends. Previous research in Australia showed, for example, that managers receive support mostly outside of the workplace, from a spouse or partner, leading them to feel “lonely at the top” (Lindorff, 2001). On the contrary, Australian nurses do benefit primarily from peers (work colleagues) support when dealing with work stress (Joiner and Bartram, 2004). While sources of social support were investigated in restricted groups (managers, nurses, students) (Urquhart and Pooley, 2007), the validation of the MSPSS can provide a measure of sources of social support for the national Australian population. Future studies can employ the MSPSS to examine the impact of these sources (i.e., significant other, family, or friends) on psychosocial outcomes (e.g., diminishing stress) at a population level, leading to targeted interventions.

Moreover, perceptions of social support are influenced by cultural differences (Glazer, 2006), including between high-income countries (Davidson et al., 2008). Hence, it is important to evaluate whether questionnaires measuring social support have appropriate functioning in distinct cultures. The need for evidence-based assessment was the reason behind the specific validations of the MSPSS (originally developed in the United States) in multiple countries and cultures (Dambi et al., 2018). The countries in which the MSPSS were validated include low-, middle-, and high-income countries, such as France (Denis et al., 2015) and Canada (Clara et al., 2003). Considering that Australia has unique sociodemographic characteristics compared to other Western high-income countries, including low population density (Pong et al., 2009) and a third of the population being born overseas (Australia Bureau of Statistics, 2016), it is also necessary to ensure that MSPSS is also valid and reliable in the Australian context.

Third, we evaluated the MSPSS psychometric properties with Bayesian confirmatory factor analysis (BCFA). Since all previous validations were conducted within a frequentist framework, the application of BCFA can provide further insight into the MSPSS psychometric properties, such as an *in-depth* evaluation of model fit through the inspection of the fit indices’ posterior distribution. The current study aims to investigate whether the MSPSS constitutes a valid and reliable measure of social support for the general Australian population.

## Methods

### Study Population

The sample comprised 3899 adult Australians in the population-based study Australia’s National Survey of Adult Oral Health (NSAOH) 2004–2006. The NSAOH sampling strategy was a three-stage (i.e., postcodes, households, people) stratified clustered design implemented to select a representative sample of the Australian population. Participants were interviewed by study staff via computer-assisted telephone interview (CATI). The participants who agreed to receive dental examinations were also mailed a questionnaire with several measures, including the MSPSS (Supplementary Table 1). Among the participants who received examination, the questionnaire response rate was 70.1% (Sanders and Slade, 2011). The NSAOH 2004–2006 was approved by the University of Adelaide’s Human Research Ethics Committee. All participants provided signed informed consent (Slade et al., 2004).

### Measures

#### The Multidimensional Scale of Perceived Social Support

The Multidimensional Scale of Perceived Social Support (MSPSS) is a 12-item instrument assessed by a 5-point scale (1 = Strongly Disagree, 2 = Disagree, 3 = Neutral, 4 = Agree, 5 = Strongly Agree). The original validation by Zimet, Dahlem (Zimet et al., 1988) indicated a three-factor structure comprising Significant Other (SO), Family (FA), and Friends (FR) subscales.

#### The Perceived Stress Scale

The Perceived Stress Scale (PSS-14) is a 14-item instrument assessed by a 5-point scale (1 = Strongly Disagree, 2 = Disagree, 3 = Neutral, 4 = Agree, 5 = Strongly Agree) with a two-factor structure of Perceived Stress (PS) and Perceived Coping (PC). A revised version has been recently validated for the Australian general population (Santiago et al., 2020).

#### Perceived Dental Control

The Perceived Dental Control (PDC-3) evaluates perceptions of control (“I don’t feel in control when I’m in the dental chair”), predictability (“I don’t feel like I know what’s going to happen next when I’m in the dental chair”), and likelihood of harm (“I believe I will be hurt when I’m in the dental chair”) when at the dentist (Armfield et al., 2008). Details of response options are as per above.

### Statistical Analysis

#### Statistical Software

The statistical analysis was conducted with R software (R Core Team, 2013) and R package blavaan 0.3-6 (Merkle and Rosseel, 2015). The Markov Chain Monte Carlo (MCMC) estimation was performed with Stan (Gelman et al., 2015) within the RStan interface (Stan Development Team, 2018). Considering that estimation with sampling weights are currently being developed for BCFA, all analyses were conducted with unweighted data. The criterion validity analysis was conducted with JASP (JASP Team, 2018).

#### Factorial Structure

The factorial structure was evaluated through BCFA (Lee, 1981). Since missing values for individual items ranged from 0.02% to 0.18%, multiple imputation was not required (Graham, 2009) and complete case analysis was conducted (*n* = 3868). The first model evaluated was the one-factor model, since it is the most parsimonious, and if it is not possible to reject a one-factor model at first, there is no need to evaluate models with a more complex factorial structure (Kline, 2015). In case the one-factor model was rejected, the next model evaluated was the two-factor structure in which the SO subscale was combined with the FR subscale, a factorial structure that has been previously reported in Chinese samples (Lai et al., 1996; Chou, 2000). For the sake of completeness, we also evaluated the two other possible two-factor models, combining the FA subscale with the FR subscale and the FA subscale with the SO subscale. Finally, we evaluated the theoretical model comprising the SO, FA, and FR factors (Zimet et al., 1988).

#### Factor Model

Let *y*_*i*_ be the *p* observed variables (OV) (i.e., observed items responses) associated with participant *i* and *m* be the number of latent variables (LV). Then, the factor model estimated was:

$$y_{i}=\nu +\Lambda \eta_{i}+\varepsilon_{i}$$

where ν is the *p* × 1 vector of intercepts for the OV, Λ is the *p* × *m* matrix of factor loadings, η_*i*_ is the m × 1 vector containing the LV such that η_*i*_ ∼ N_m(0,ϕ) and ε_*i*_ is the *p* × 1 vector of residuals distributed as ε_*i*_∼*N*_*p*(0,∑). In addition, ε_*i*_ and η_*i*_ were assumed to be uncorrelated. The LV were assumed to covary, so ϕ is the m × m latent variable covariance matrix (Jöreskog and Sörbom, 1996; Merkle and Rosseel, 2015). The graphical representation of the structural equation models (SEMs) is displayed in Supplementary Figure 1.

The factor models were estimated with a mean structure (i.e., intercept parameters), originally developed for continuous items. Although factor models with a threshold structure can also be estimated in BCFA (Lee, 2007), there is one major limitation that withholds its implementation in the current study. The only fit index currently available for factor models with a threshold structure is the χ^2^ statistic in which the null hypothesis represents the *exact* correspondence between the model-implied covariance matrix and sample covariance matrix (Gelman et al., 1996; Sellbom and Tellegen, 2019; Taylor, 2019). Fit indices, such as the RMSEA and the comparative fit index (CFI) (Bentler, 1990), which were developed to complement the χ^2^ statistic and describe the *degree* of correspondence between the model and the data (Garnier-Villarreal and Jorgensen, 2019), have only been validated for factor models with a mean structure in BCFA (Garnier-Villarreal and Jorgensen, 2019; Hoofs et al., 2018). Although factor models with a threshold structure are potentially more aligned with the ordered-categorical nature of MSPSS items, interpretation of model fit would be restricted using these models. That is, under factor models with a threshold structure, we would only be able to evaluate the exact correspondence between the model and the data using the χ^2^ statistic, and it is unlikely that any hypothesized factorial structure can *exactly* reproduce the MSPSS item responses (MacCallum, 2003). For this reason, factor models with a mean structure were estimated. Finally, R package *blavaan* can fit only factor models with a mean structure (instead of a threshold structure) in its current version.

#### Model Estimation

Model estimation was carried out with three independent MCMC chains with Hamiltonian Monte Carlo sampling (Duane et al., 1987). The estimation was performed with 1000 iterations for each chain after a burn-in period of 1000 iterations. Convergence of the MCMC chains to the posterior distribution was evaluated graphically with (a) trace plots (Gelfand and Smith, 1990) and formally with (b) the estimated potential scale reduction factor (PSRF) (Gelman and Rubin, 1992) and the (c) the Monte Carlo standard error (MCSE) using batch means (Jones et al., 2006). PSRF values for each parameter close to 1.0 indicate convergence to the posterior distribution (Brooks and Gelman, 1998). Brooks and Gelman (Brooks and Gelman, 1998) recommended that when PSRF values for each parameter are close to 1.0 and smaller than 1.1, convergence to the posterior distribution can be considered to be reached. Otherwise, MCMC chains with more iterations are necessary to improve convergence to the posterior distribution.

Vague priors [default in blavaan (Merkle and Rosseel, 2015) for estimation using Stan] were specified for the factor loadings [λ∼ N(μ = 0, σ^2^ = 100)], OV intercepts [ν∼ N(μ = 0, σ^2^ = 1024)], OV residual standard deviations [ε∼ G(1,0.50)], and LV residual standard deviations [ε∼ G(1,0.50)]. Each factor correlation had a prior uniform distribution on the interval [−1, 1]. The parameters were, *a priori*, assumed to be mutually independent (Scheines et al., 1999). Recent simulation studies have shown this set of priors to be weakly informative for a variety of SEMs typically encountered in practice (Merkle et al., 2020). When the sample size is large (or very large) and vague priors are specified, the posterior distribution is predominantly informed by the likelihood function, and results become asymptotically equal to a maximum likelihood (ML) solution (Garnier-Villarreal and Jorgensen, 2019). To illustrate this equivalence, we reported results from maximum likelihood estimation in the Supplementary Material. The latent variables were scaled using the reference variable method, imposing a unit loading identification (ULI) constraint on the first item of each subscale (Kline, 2015). Completely standardized solutions of the factor analytical models were reported.

#### Model Fit

Model fit was investigated through posterior predictive model checking (PPMC) (Gelman et al., 1996). PPMC uses a discrepancy function to calculate whether the observed data are consistent with the expected values of the model at each iteration of the Markov chain that successfully converged to the posterior distribution. In our study, the discrepancy function selected was the χ^2^ statistic, which compares the sample covariance matrix (S) with the model-implied covariance matrix ($\hat{\sum }$) (Gierl and Mulvenon, 1995). The χ^2^ statistic is displayed below:

$$\chi^{2}=N(log|\hat{\Sigma }|-log|S|+trace\left(S{\hat{\Sigma }}^{-1}\right)$$

$$-p+{\left(\bar{y}-\hat{\mu }\right)}^{T}{\hat{\Sigma }}^{-1}\left(\bar{y}-\hat{\mu }\right))$$

where $\bar{y}$ is the *p* × 1 vector of sample means, and $\hat{\mu }$ is the *p* × 1 vector of model-implied means. The fit of the SEM was then evaluated with the posterior predictive *p*-value (PPP_χ_2). The PPP_χ_2 estimates the proportion of posterior samples from which the discrepancy measure calculated with observed data (D^*obs*^) is higher than the discrepancy measure calculated with replicated data (D^*rep*^) under the model. The rationale is that, if the observed data is *perfectly* explained by the model, occasions when D^*obs*^ > D^*rep*^ (or D^*obs*^ < D^*rep*^, for that matter) are arbitrary and the PPP_χ_2 should approximate 50% (Garnier-Villarreal and Jorgensen, 2019).

The limitation of the PPP_χ_2 is that the χ^2^ statistic evaluates the null hypothesis of *exact* correspondence between the model-implied covariance matrix and sample covariance matrix. However, theoretical models, such as the MSPSS three-factor structure comprising SO, FA, and FR (Zimet et al., 1988), were created to be merely *approximations* of reality and were not expected to perfectly explain observed data from empirical research (MacCallum, 2003). Sellbom and Tellegen (Sellbom and Tellegen, 2019) emphasize that, in psychological assessment research with factor analysis, “the null hypothesis is virtually always rejected, which means that there will always be significant discrepancies between the estimated model parameters and observed data.” Thus, as the sample size increases, the χ^2^ statistic becomes more and more sensitive to detect trivial deviations from the model. This limitation of the χ^2^ statistic is present in both frequentist and Bayesian CFA and has been reiterated by several methodologists (Saris et al., 2009; Asparouhov and Muthén, 2010; West et al., 2012; Hayduk, 2014; Garnier-Villarreal and Jorgensen, 2019). Hence, when the study has enough power, the PPP_χ_2 will detect trivial model misspecifications, even when these misspecifications have no substantive or practical meaning. While more studies are needed, the sensitivity of the PPP_χ_2 to detect negligible differences within large samples seems to approach 1.0 (Hoofs et al., 2018), requiring other fit indices such as RMSEA and CFI to be used to evaluate model fit.

For this reason, we also evaluated the fit of the model with indices such as the RMSEA and the CFI, which complement the χ^2^ statistic by indicating the degree of correspondence between the model and the data (Garnier-Villarreal and Jorgensen, 2019). Based on previous work by Hoofs et al. (2018), Garnier-Villarreal and Jorgensen (2019) recently adapted fit indices to Bayesian structural equation modeling. The proposed Bayesian RMSEA (BRMSEA) and Bayesian CFI (BCFI) are displayed below:

$${\mathrm{BRMSEA}}_{i}^{\mathrm{DevM}}=\sqrt{\text{max}\left[0,\frac{D_{obs}-p\text{*}}{\left(p\text{*}-pD\right)*N}\right]}$$

$${\mathrm{BCFI}}_{i}^{\mathrm{DevM}}=1-\frac{D_{H,i}^{obs}-p\text{*}}{D_{0,i}^{obs}-p\text{*}}$$

where *p*^∗^ is the number of unique elements within the sample variance–covariance matrix, *i* is the Markov chain iteration, *N* is the sample size, $D_{H,i}^{obs}$ is the χ^2^ statistic (previously described) calculated with observed data (D^*obs*^) under the hypothesized model, $D_{0,i}^{obs}$ is the χ^2^ statistic calculated with observed data (D^*obs*^) under the independence model, and pD is the *effective* number of parameters. Since the number of parameters in Bayesian inference cannot be expressed as integers (e.g., informative compared to noninformative priors further restrict the parameter space), we used the effective number of parameters (pD) based on the deviance information criteria (DIC) (Spiegelhalter et al., 2002). The pD was calculated through the marginalized DIC (mDIC) after latent variables were integrated out (Quintero and Lesaffre, 2018). The independence model was specified by constraining covariances among observed variables to zero and freely estimating intercepts and variances (Widaman and Thompson, 2003).

In terms of interpretation, since widely used cutoff points derived from frequentist simulation studies (Hu and Bentler, 1999) do not provide the same type I and II error rates in BSEM, hypothesis testing using these cutoffs with BRMSEA and BCFI should not be conducted. Nonetheless, Garnier-Villarreal and Jorgensen (Garnier-Villarreal and Jorgensen, 2019) explained that “traditional guidelines proposed for interpreting the magnitude of SEM fit indices based on intuition and experience would be no less valid.” For this reason, we evaluated the *magnitude* of fit indices such as BRMSEA and BCFI as descriptive measures of the degree to which the model failed to reproduce the data (Garnier-Villarreal and Jorgensen, 2019).

#### Dimensionality

In addition to the evaluation of model fit of the one-, two-, and three-factor models, we used the Bayes factor (BF) (Jeffreys, 1961) to *formally* test which factorial structure has more support from the data. The BF compares the marginal likelihood of the data under model 2 (the *alternative hypothesis)* with the marginal likelihood of the data under model 1 (the *null hypothesis*), providing thus a continuous measure of the predictive accuracy of the two competing models (Wetzels and Wagenmakers, 2012). Since calculation of the BF can be difficult, the log-Bayes factor (logBF) was calculated using the Laplace approximation (Lewis and Raftery, 1997) and then converted to the BF.

*Reliability.* Similarly, we propose calculating reliability using a Bayesian version of McDonald’s Ω_*H*_ (McDonald, 2013):

$$B\Omega_{H}=\frac{1}{\left(1^{\prime }\Sigma 1\right)s}\Sigma_{i}{\left(\Sigma_{j=1}^{k}\lambda_{i}^{j}\right)}^{2}Var\left(\psi_{i}\right)$$

where $\lambda_{i}^{j}$ is the factor loading of item *j*, *i* is the Markov chain iteration, *s* is the number of Markov chain iterations, ψ is the factor variance, Σ is the sample covariance matrix, and **1** is the *k*-dimensional vector of 1’s. The McDonald’s Ω_*H*_ was chosen over traditional reliability such as Cronbach (1951)α, since it does not assume (1) tau equivalence or a (2) congeneric model without correlated uniqueness (Dunn et al., 2014).

#### Criterion Validity

Nonparametric bivariate Kendall’s τ (Kendall, 1948) correlations were calculated between the MSPSS subscales total score (SO, FA, and FR), subscales total scores from the Perceived Stress and Perceived Coping (PSS), and total scores from the PDC. Since the PSS subscale total scores and the PDC total scores had missing values, we employed pairwise exclusion to calculate the Kendall’s τ correlations with the MSPSS subscales total score. For the Kendall’s τ correlations, the prior employed was also vague. The prior for the Kendall’s τ correlation is a uniform distribution on the interval [−1, 1] (Van Doorn et al., 2018; Wagenmakers et al., 2018a). Perceived stress was chosen for the evaluation of criterion validity since a large body of empirical research has provided evidence of the protective effects of social support on stress (Lakey and Cohen, 2000). Hence, it was expected a negative correlation of the MSPSS subscales with Perceived Stress (convergent validity) and a positive correlation with Perceived Coping (divergent validity). We also expected a weak and nonmeaningful correlation (discriminant validity) between social support and perceived control during dental examination (measured by the PDC-3). The appointment with the dentist occurs on the dental examination room, where individuals undergo examination alone (without a significant other, family, friends). Hence, the PDC-3 evaluates perceptions of control limited to the experience of dental examination (e.g., “I don’t feel like I know what’s going to happen next when I’m in the dental chair”), and these perceptions were shown to be more associated with personality factors (neuroticism, trait anxiety) (Brunsman et al., 2003) than the social support received in other domains of life.

### Advantages Over Frequentist ML

In our study, CFA with Bayesian inference and evaluation of fit with BRMSEA and BCFI were chosen due to three main advantages over the frequentist approach. First, (1) fit indices such as CFI are complex functions of model parameters and have unknown sampling distributions precluding the calculation of *precision* measures such as 95% confidence intervals (CI). Although the RMSEA is an exception since it has a known sampling distribution (Browne and Cudeck, 1992), the BCFI and BRMSEA provide an empirical approximation of the “*realized* values of the discrepancy measure” (Levy, 2011) for any sample size without the need to rely on asymptotic theory. Second, (2) the interpretation of credible intervals (CrI) (i.e., 95% probability that the *true* parameter value lies between the interval) is more intuitive than the interpretation of CI since statistical inference is conducted by conditioning *on the study data* rather than depending on infinite repetitions of the estimator (Morey et al., 2016). Third, (3) hypothesis testing with *p* values has received strong criticism over the decades (Cohen, 1994). *P*-values are considered a confounded measure since they depend upon both effect size *and* sample size (Lang et al., 1998). Moreover, the failure to reject the null hypothesis does not prove the null is correct or preferable than an alternative hypothesis (Wagenmakers, 2007), so “absence of evidence is not evidence of absence” (Jaykaran et al., 2011). Finally, authors such as Amrhein, Greenland (Amrhein et al., 2019) recently emphasized that the use of cutoff points and dichotomization of *p-*values into “significant” and “nonsignificant” should be *abandoned*, since similar effect sizes with *p-*values below and above thresholds (e.g., above and below 0.05) should not be interpreted differently. Therefore, all statistical hypothesis tests in the current study were conducted with the BF.

Although the current study had a large sample, another advantage of BCFA is in small sample sizes, in which frequentist estimation often results in nonconvergence and inaccurate parameter estimates (Smid et al., 2020). The reason is that frequentist procedures rely on “asymptotic results that are typically not satisfied with psychometric data except in large-scale settings” (Rupp et al., 2004). However, in psychological research, many target populations can be naturally small or hard to access groups (e.g., burn survivor patients with posttraumatic stress symptoms) (Van De Schoot et al., 2015), so recruiting large samples is not feasible or even possible. In these cases, BCFA can also provide a powerful alternative to frequentist CFA. Thus, future validations of the MSPSS targeted at small, selected populations would also benefit from BCFA.

## Results

### Demographic Characteristics

The age range of participants was 18–82 years (*M*_*age*_ = 50.2, *SD* = 14.8); almost two-thirds were women (62.1%), over two-thirds had received a tertiary education (67.4%), and almost 60% were employed (Table 1). There were no meaningful differences between the original sample (*n* = 3899) and the complete cases sample (*n* = 3868).

**TABLE 1:** Participants characteristics.

	Original sample (n = 3899)		Complete cases (n = 3868)	
	N	%	n	%
**Age**				
Mean	50.2			50.1		
SD	14.8			14.8		
Min/Max	18–82			18–82		
Missing	0	0%	0	0%
**Sex**				
Female	2420	62.1%	2398	62.0%
Male	1479	37.9%	1470	38.0%
Missing	0	0%	0	0%
**Education**				
High school or less	1272	32.6%	1256	32.5%
TAFE or university	2627	67.4%	2612	67.5%
Missing	0	0%	0	0%
**Employed**				
Yes	2299	59.0%	2288	58.7%
No	1600	41.0%	1580	40.8%
Missing	0	0%	0	0%

*Note: TAFE, Technical and Further Education (trade school/college).*

### Model Convergence

In all models evaluated in this study, the one-, two-, and three-factor models, the Markov chains converged to the posterior distribution with 1000 iterations after discarding the first 1000 iterations as a burn-in. The visual inspection of trace plots indicated convergence of the three Markov chains. Trace plots of the three-factor model are reported (Supplementary Figure 2). The PSRF for individual parameters were very close to 1.00 and smaller than 1.10 in all models. The PSRFs of the three-factor model are displayed in Table 2, while the PSRFs of the one- and two-factor models are displayed in Supplementary Tables 2–5.

**TABLE 2:** Model parameters of the three-factor model.

	Posterior mean	95% CrI	PSRF
***Factor Loadings—Significant Other***			
There is a special person who is around when I am in need.	0.898	[0.890, 0.905]	–
There is a special person with whom I can share joys and sorrows.	0.936	[0.930, 0.941]	1.001
I have a special person who is a real source of comfort to me.	0.908	[0.901, 0.915]	1.000
There is a special person in my life that cares about my feelings.	0.873	[0.864, 0.882]	1.000
***Factor Loadings—Family***			
My family really tries to help me.	0.866	[0.856, 0.875]	-
I get the emotional help and support I need from my family.	0.924	[0.917, 0.931]	1.000
I can talk about my problems with my family.	0.821	[0.809, 0.832]	0.999
My family is willing to help me make decisions.	0.829	[0.817, 0.839]	1.000
***Factor Loadings—Friends***			
My friends really try to help me.	0.862	[0.852, 0.872]	-
I can count on my friends when things go wrong.	0.884	[0.874, 0.892]	0.999
I have friends with whom I can share my joys and sorrows.	0.881	[0.871, 0.890]	0.999
I can talk about my problems with my friends.	0.841	[0.830, 0.852]	1.000
***Intercepts***			
There is a special person who is around when I am in need.	4.048	[3.395, 4.174]	1.000
There is a special person with whom I can share joys and sorrows.	4.272	[4.167, 4.367]	1.000
I have a special person who is a real source of comfort to me.	3.979	[3.885, 4.080]	0.999
There is a special person in my life that cares about my feelings.	4.126	[4.028, 4.227]	1.000
My family really tries to help me.	4.291	[4.138, 4.386]	1.001
I get the emotional help and support I need from my family.	4.037	[3.943, 4.133]	1.001
I can talk about my problems with my family.	3.883	[3.792, 3.977]	1.001
My family is willing to help me make decisions.	4.072	[3.971, 4.169]	1.001
My friends really try to help me.	4.546	[4.451, 4.657]	1.000
I can count on my friends when things go wrong.	4.430	[4.330, 4.538]	1.000
I have friends with whom I can share my joys and sorrows.	4.538	[4.432, 4.644]	1.000
I can talk about my problems with my friends.	4.249	[4.157, 4.352]	1.000
***Residual variances***			
There is a special person who is around when I am in need.	0.194	[0.181, 0.207]	1.000
There is a special person with whom I can share joys and sorrows.	0.124	[0.113, 0.134]	0.999
I have a special person who is a real source of comfort to me.	0.176	[0.163, 0.189]	0.999
There is a special person in my life that cares about my feelings.	0.238	[0.222, 0.253]	0.999
My family really tries to help me.	0.250	[0.234, 0.253]	0.999
I get the emotional help and support I need from my family.	0.146	[0.133, 0.159]	0.999
I can talk about my problems with my family.	0.326	[0.307, 0.346]	0.999
My family is willing to help me make decisions.	0.314	[0.295, 0.331]	0.999
My friends really try to help me.	0.257	[0.240, 0.274]	0.999
I can count on my friends when things go wrong.	0.219	[0.203, 0.235]	0.999
I have friends with whom I can share my joys and sorrows.	0.224	[0.208, 0.241]	0.999
I can talk about my problems with my friends.	0.292	[0.274, 0.311]	0.999
*Significant Other*	1.000	[1.000, 1.000]	1.000
*Family*	1.000	[1.000, 1.000]	1.000
*Friends*	1.000	[1.000, 1.000]	1.000
***Factor correlations***			
*Significant Other∼∼Family*	0.636	[0.614, 0.658]	0.999
*Significant Other∼∼Friends*	0.505	[0.479, 0.531]	1.000
*Family∼∼Friends*	0.537	[0.512, 0.562]	0.999

*Note: There are no PSRFs for Item 1, Item 3 and Item 6 since unstandardized factor loadings were constrained to one during estimation (unit loading identification). CrI, credible interval; PSRF, potential scale reduction factor.*

The MCSEs of the one-factor model ranged from 9.49 × 10^–5^ to 9.63 × 10^–4^, the MCSEs of the two-factor model ranged from 4.06 × 10^–19^ to 9.63 × 10^–4^, and the MCSEs of the three-factor model ranged from 4.23 × 10^–5^ to 9.73 × 10^–4^. In all cases, the MCSEs of the parameters were smaller than 5% of the posterior standard deviation.

### Factorial Structure

The evaluation of PPP_χ_2 across all models indicated that the χ^2^ statistic, which evaluates null hypothesis of exact correspondence between the model-implied covariance matrix and sample covariance matrix, calculated with observed data (D^*obs*^) was substantially higher when compared to the χ^2^ statistic calculated with replicated data (D^*rep*^) (PPP_χ_2 < 0.001). Considering that the PPP_χ_2 can be sensitive to trivial model misspecifications under large samples (as the sample in our study), we proceeded then to inspect fit indices such as RMSEA and CFI to evaluate the degree of correspondence between the model and the data. The first model evaluated was the one-factor model and it displayed a poor fit (Table 3).

**TABLE 3:** Fit statistics for the factor analytical models.

Model	χ^2^ (Posterior mean)	*pD*	*df*	PPP_χ2_	BRMSEA	90% CrI	BCFI	90% CrI
**Bayesian CFA**								
*One-factor structure*	17,344.41	35.78	54.21	< 0.001	0.287	[0.287, 0.288]	0.596	[0.595, 0.596]
*Two-factor structure (FR and SO)*	10,867.80	36.70	53.29	< 0.001	0.229	[0.228, 0.229]	0.747	[0.746, 0.747]
*Two-factor structure (FA and FR)*	9,908.214	37.11	52.89	< 0.001	0.219	[0.219, 0.220]	0.769	[0.769, 0.770]
*Two-factor structure (FA and SO)*	8,886.30	36.70	53.30	< 0.001	0.207	[0.206, 0.207]	0.793	[0.793, 0.794]
*Three-factor structure (SO, FA and FR)*	1,609.87	39.09	50.91	< 0.001	0.089	[0.088, 0.089]	0.963	[0.963, 0.964]

*Note: CFA, confirmatory factor analysis; χ^2^, chi-square; pD, effective number of parameters; df, degrees of freedom; PPP_χ_2, posterior predictive p-value; BRMSEA, root mean square error of approximation; CrI, credible interval; BCFI, Bayesian comparative fit index.*

**FIGURE 1:**
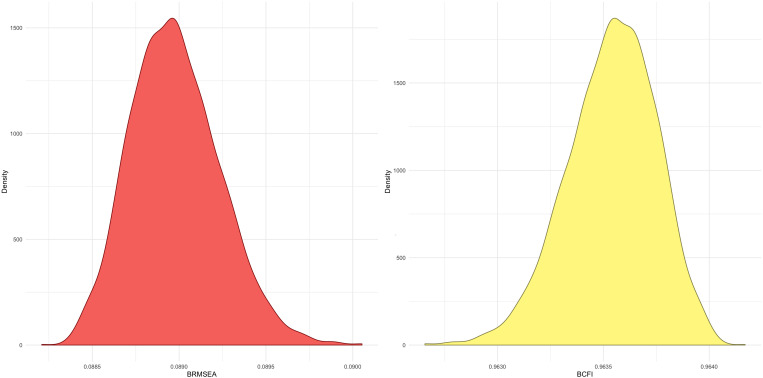
Kernel density plots of the realized posterior distributions of approximate fit indices. Note: The realized posterior distribution of the Bayesian root mean square error of approximation (BRMSEA) (left plot) and Bayesian comparative fit index (BCFI) (right plot) are displayed.

All the two-factor models, such as the two-factor model in which the SO and FR subscales were combined, also displayed poor fit. The theoretical three-factor model (SO, FA, and FR) was evaluated, and the fit to the data was substantially improved. ML estimation results are shown in Supplementary Table 6 by means of comparison. The inspection of the three-factor model BRMSEA’s posterior distribution indicated values consistent with an adequate model fit, while the BCFI’s posterior distribution comprised values consistent with a good fit (Figure 1).

Furthermore, all factor loadings evaluated at the posterior mean were higher than 0.8, and high factor loadings (> 0.80) were observed across the entirety of the posterior distributions, as indicated by the 95% credible intervals (Table 2). For these reasons, the three-factor structure was confirmed as an adequate measurement model for the MSPSS.

### Dimensionality

The BF indicated decisive support for the three-factor model in comparison with the one-factor model (BF_31_ = 5.94 × 10^3409^) and in comparison with all two-factor models. For instance, the BF indicated decisive support for the three-factor model in comparison with the two-factor model in which SO and FR subscales were combined (BF_32_ = 9.22 × 10^2005^).

### Reliability

The SO [BΩ_*H*_ = 0.95 - 95% CrI (0.90, 0.99)], FA [BΩ_*H*_ = 0.92 - 95% CrI (0.87, 0.97)], and FR [BΩ_*H*_ = 0.92 - 95% CrI (0.88, 0.97)] subscales displayed excellent reliability.

### Criterion Validity

The Kendall’s τ correlations between the MSPSS subscales and the Perceived Stress subscale, Perceived Coping subscale, and PDC-3 are displayed in Table 4. The MSPSS subscales displayed weak positive correlations with the Perceived Stress subscale and weak negative correlations with the Perceived Coping subscale.

**TABLE 4:** Criterion validity of the Multidimensional Scale of Perceived Social Support.

				90% CrI	
Subscales	N	Kendall’s τ	BF10	Lower	Upper
Significant Other—Perceived Stress	3862	−0.164	1.23 × 10^49^	−0.182	−0.147
Family—Perceived Stress	3862	−0.219	4.47 × 10^89^	−0.237	−0.202
Friends—Perceived Stress	3862	−0.149	2.04 × 10^40^	−0.167	−0.132
Significant Other—Perceived Coping	3855	0.219	2.72 × 10^88^	0.201	0.236
Family—Perceived Coping	3855	0.246	1.90 × 10^112^	0.228	0.263
Friends—Perceived Coping	3855	0.204	7.65 × 10^76^	0.186	0.222
Significant Other—Perceived Dental Control	3841	−0.039	14.31	−0.057	−0.022
Family—Perceived Dental Control	3841	−0.078	5.60 × 10^9^	−0.096	−0.061
Friends—Perceived Dental Control	3841	−0.084	4.38 × 10^11^	−0.102	−0.067

*Note: BF10, Bayes factor of the alternative hypothesis over the null hypothesis of zero correlation. Bayes factor values that are larger than one favor the alternative hypothesis of nonzero correlation. CrI, credible interval.*

Furthermore, there was no meaningful association between the SO, FA, and FR subscales and the PDC-3. Although the Bayes factor provided strong support for the alternative hypothesis of nonzero correlation between SO and PDC-3 and decisive support for the alternative hypothesis of nonzero correlation between FA and PDC-3, the magnitudes of these correlations were close to zero, indicating that these correlations were negligible and had no practical meaning.

## Discussion

The present study aimed to evaluate whether the MSPSS constitutes a valid and reliable instrument to measure social support in a large sample of non-Aboriginal Australians. The findings confirmed that the theoretical three-factor structure composed of SO, FA, and FR and reliability was excellent. The implications for future use of the MSPSS in Australia are discussed.

### Factorial Structure

The findings provided support for the theoretical three-factor model composed by SO, FA, and FR. While the fit of the one- and two-factor models were unacceptable, the three-factor model provided a good fit to the data.

In the three-factor model, the very small PPP_χ_2 (< 0.001) indicated that compared to the replicated data *under the model*, the observed data consistently showed stronger discrepancies with respect to the model. Authors such as Saris, Satorra (Saris et al., 2009) and West, Taylor (West et al., 2012) have argued that such discrepancies are expected and unavoidable in the light of the large sample sizes needed for sufficient statistical power to estimate SEM model parameters (Sellbom and Tellegen, 2019). That is, the PPP_χ_2 can detect trivial discrepancies that have no substantive meaning, even when the theoretical model (for example, the MSPSS structure of SO, FA, and FR) constitute a good approximation of reality (Garnier-Villarreal and Jorgensen, 2019). In Bayesian CFA, the PPP_χ_2 sensitivity to detect negligible differences increases with sample size and seems to approach 100% under large samples (Hoofs et al., 2018). Thus, the very small PPP_χ_2 (< 0.001) observed for the three-factor model does not indicate poor fit of the three-factor model by itself and needs to be complemented and considered with the other fit indices such as BRMSEA and BCFI.

For instance, the BRMSEA of the three-factor model was within “traditional guidelines proposed for interpreting the magnitude of SEM fit indices” (Garnier-Villarreal and Jorgensen, 2019), such as that “a value of about 0.08 or less for the RMSEA would indicate a reasonable error of approximation” (Browne and Cudeck, 1992). Similarly, the BCFA was above the usually recommended value of 0.95 (Yu, 2002). In the end, despite the small PPP_χ_2 (< 0.001), the BRMSEA and BCFI clearly indicated adequate model fit of the three-factor structure. When the models were compared, the BF provided decisive support for the three-factor structure in relation to the other two structures. For example, the BF suggested that the data are 3.90 × 10^1991^ times more likely to occur under the three-factor structure compared to the two-factor structure in which the SO and FR subscales were combined.

The support for the three-factor structure is consistent with the literature and indicates that significant other, family, or friends are *independent* sources, which provide *qualitatively distinct* social support. For example, it is known that social support from a significant other, such as a romantic partner, is particularly relevant when an individual is facing unemployment. In this situation, social support from a partner can increase the perception that striving to pursue a job is a worthwhile endeavor (Vinokur and Caplan, 1987). Alternatively, support from friends can be especially relevant in the face of relationship stress. When an individual is experiencing problems within a relationship, a friend can become a confidant and provide advice due to not being directly involved in the relationship dynamics (Jackson, 1992). In the current study, the correlations between SO, FA, and FR subscales ranged from 0.50 to 0.64, showing that these dimensions were moderately correlated but without posing concerns regarding discriminant validity (*r* > 0.80) (Brown, 2014). That is, the dimensions were correlated (e.g., individuals who received support from family also reported receiving support from friends and a significant other), but the sources of support were distinct (e.g., some individuals received more support from family than from other sources, such as friends and a significant other). For this reason, total scores should be computed for each subscale (SO, FA, and FR) independently instead of a total score computed based on all 12 items.

The two-factor model in which the SO and FR subscales were combined was not a good fit for the data, indicating that Australian respondents did discriminate between the terms “special person” and “friends.” In the current study, the MSPSS was applied in its original format (Zimet et al., 1988) without any additional explanations to the “special person” term such as “a girlfriend/boyfriend, fiancé, relative, neighbour, or a doctor” (Eker et al., 2001). Therefore, considering that the majority of Australians have English as their native language (McDonald et al., 2019), the original MSPSS can be applied in Australia without revisions. Moreover, the two other possible two-factor models, in which FA and FR or FA and SO were combined, also did not show good fit to the data, indicating that a three-factor structure better explained the item responses in the Australian population.

### Reliability

The reliability of the three subscales was excellent, consistent with previous MSPSS psychometric studies (Pushkarev et al., 2018). Despite not being yet subjected to simulation studies, we proposed a Bayesian version of Ω_*H*_ by calculating the Ω_*H*_ formula at each iteration of the Markov chain, which creates a posterior distribution for the BΩ_*H*_ statistic. Since under uninformative priors the mean of the posterior distribution should approximate the maximum likelihood estimate, the BΩ_*H*_ expectedly resembled the Ω_*H*_. In our study, the BΩ_*H*_ and ML Ω_*H*_ were equivalent to a three-decimal precision. The approach we took was different from Yang and Xia (2019), who recently evaluated Bayesian estimation of the categorical Ω_*H*_ by substituting “central tendency measures such as the medians of the posterior distributions” into the original categorial Ω_*H*_ formula.

### Criterion Validity

The SO, FA, and FR subscales displayed the expected patterns of convergent and divergent validity since they were negatively correlated with Perceived Stress and positively correlated with Perceived Coping. These associations were consistent with research evidence showing that social support is protective against stress (Lakey and Cohen, 2000) since it provides external resources to overcome stressful events and promotes individual coping by enhancing feelings of personal control (Lincoln et al., 2003). Although the magnitudes of these correlations were weak, a recent systematic review by Harandi, Taghinasab (Harandi et al., 2017) showed that social support is only moderately correlated with mental health outcomes. Moreover, since social support and perceived stress/coping are theoretically related but qualitatively distinct constructs, correlations with small magnitudes have also been previously reported (Hamdan-Mansour and Dawani, 2008; Santiago et al., 2019). In general, the observed correlations in our study provided initial support for the MSPSS convergent and divergent validity, but future studies should further investigate the MSPSS convergent and divergent validity in Australia.

Regarding discriminant validity, the SO, FA, and FR subscales displayed close to zero correlations with the PDC-3. Accordingly, it was expected that social support would be weekly associated with perceived control during dental examination. Although the presence of a significant other, family, or friends in the dental clinic is potentially beneficial (Bernson et al., 2011), previous research had emphasized individual characteristics, such as personality traits (Brunsman et al., 2003) and genetic vulnerability (Carter et al., 2014), as leading factors impacting perceived control during examination by the dentist.

### Sample

Although good psychometric properties have previously been reported, to the best of our knowledge, this study was the first to evaluate the validity of the MSPSS for a large, general heteregenous population. The findings showed that the MSPSS can adequately measure perceptions of social support according to different sources of support (significant other, family, and friends) at a national level in Australia. Thus, the MSPSS can be included in national surveys and applied in future large studies conducted in the Australian context.

### Strengths and Limitations

The current study had several strengths, such as the use of modern BSEM methodology to conduct the psychometric analysis. Despite resources such as the modification index (Sörbom, 1989) and fit indices for multigroup CFA (Cheung and Rensvold, 2002) used in frequentist analysis being currently under development for BSEM, the inferences based on the entirety of the posterior distribution provided substantial advantages to the comprehension of the MSPSS psychometric functioning. For example, BSEM enabled us to evaluate the (“realized”) posterior distribution of the CFI and certify that all values were congruent with a good fitting model.

The same reasoning was possible regarding the strength of factor loadings and factor correlations. For instance, the examination of an MSPSS factor loading 95% credible interval informed that there is a 95% probability that the true factor loading in the population lied between that upper and lower limit. Since this probabilistic interpretation is naturally intuitive for researchers, clinicians, and policymakers, it has also been commonly (and erroneously) attributed to frequentist 95% confidence intervals (Morey et al., 2016). However, even in circumstances where 95% credible intervals and 95% confidence intervals are *numerically* similar, “they are not mathematical equivalent and conceptually quite different” (Van de Schoot et al., 2014). In summary, the investigation of MSPSS parameters through the posterior distribution and 95% credible intervals provides a more intuitive interpretation for researchers and policymakers, providing statements about precision and plausibility (rather than fixed long-term probabilities) (Morey et al., 2016), about the MSPSS psychometric properties.

Moreover, Bayesian estimation and hypothesis testing with the BF provides “a practical solution to the pervasive problems of *p*-value” (Wagenmakers, 2007) in psychometric research. In our study, the use of BF was relevant for comparing the MSPSS competing factorial structures. In a recent systematic review conducted by Dambi, Corten (Dambi et al., 2018) regarding MSPSS validations across multiple cultures, one main criticism was that the majority of studies used EFA and did not adequately describe fit indices or compare alternative structures (i.e., one-, two-, and three-factor models). Among the previous studies that did employ CFA to compare the MSPSS factorial structures, support for the three-factor model was provided (Clara et al., 2003; Stewart et al., 2014). While CFA fit indices (Golino et al., 2020) or information criterion such as the Akaike Information Criterion (AIC) (Akaike, 1987) or Bayesian Information Criterion (BIC) (Schwarz, 1978) can be used as relative measures of fit, the advantage of the BF is that it allows for a *direct* comparison between two competing models. That is, the BF provides a clear interpretation of how many times the evidence favors one model over the other (Wagenmakers et al., 2018b). In the case of the MSPSS, our findings concurred with previous studies that the three-factor should be preferred (Clara et al., 2003; Stewart et al., 2014) but also provided new evidence on *how many times* the data favored the MSPSS three-factor structure over other models. The BF showed, for instance, that the evidence towards the three-factor model compared to the two- and one-factor models in Australia was extreme (Jeffreys, 1961; Lee and Wagenmakers, 2014).

The study also had limitations. First, the data were from a survey conducted in 2004–2006, so over the last decade, the distribution of social support in the Australian population may have changed. Therefore, future studies should investigate whether the functioning of items remained stable or there was *item parameter drift* (Goldstein, 1983). Second, only two measures (the PSS and PDC-3) were used for the analysis of criterion validity and we could not provide strong evidence of the MSPSS external validity. Future studies should investigate convergent, discriminant, and predictive validity of the MSPSS in Australia more broadly and using other selected measures. Third, estimation with sampling weights are under development for BCFA, so psychometric analyses were conducted in the unweighted sample, which, despite constituting a large sample of Australian adults, it is not representative of the Australian population. Finally, fit indices such as RMSEA and CFI for factor models with a threshold structure, models originally developed for ordered-categorical items (Muthén, 1984), have not yet been validated for BCFA (Yu, 2002). Hence, the application of factor models with mean structure to MSPSS items limits the investigation of all possible parameters of interest, such as *threshold* parameters. *Threshold* parameters indicate the amount of a *latent response variable* that, when exceeded, predict the preference for one response category (e.g., Strongly Agree) over another (e.g., Agree) (Kline, 2015). Once these fit indices are validated for threshold models in BCFA and its calculation made available in state-of-the-art software, future studies should further investigate the MSPPS using these models.

## Conclusion

The good psychometric properties and excellent reliability of the MSPSS were confirmed in a large sample of Australian adults. The MSPSS comprised three subscales, Significant Other, Family, and Friends. Total scores should be computed for each subscale independently. Furthermore, the MSPSS can be applied at a national level, including in national surveys. The MSPSS test scores can disclose important importation regarding the sources of social support in Australia and provide evidence to the role of social support in the Australian population.

## Data Availability Statement

The datasets generated for this study are available on request to the corresponding author.

## Ethics Statement

The studies involving human participants were reviewed and approved by University of Adelaide’s Human Research Ethics Committee. The patients/participants provided their written informed consent to participate in this study.

## Author Contributions

PS, AQ, LS, RR, and LJ conceptualized the project. PS and AQ conducted the formal analysis. LJ provided resources and funding acquisition. PS, AQ, and DH wrote the initial version of the manuscript. PS, LS, RR, LJ, DH, and AQ interpreted the data. LS, RR, LJ, DH, and AQ provided theoretical and statistical supervision. All authors reviewed and edited the final draft.

## Conflict of Interest

The authors declare that the research was conducted in the absence of any commercial or financial relationships that could be construed as a potential conflict of interest.
