# Supplementary material for: Drop-the-p: Bayesian CFA of the Multidimensional Scale of Perceived Social Support in Australia
Source: Front Psychol. 2021 Feb 26;12:542257. doi: 10.3389/fpsyg.2021.542257 (PMC7953044; doi:10.3389/fpsyg.2021.542257)
Supplement: Supplementary file 1 [file Table_1.docx]

Supplementary Table 1. The MSPSS items.

| Item number | Item content |
| --- | --- |
| 1 | There is a special person who is around when I am in need. |
| 2 | There is a special person with whom I can share joys and sorrows. |
| 3 | My family really tries to help me. |
| 4 | I get the emotional help and support I need from my family. |
| 5 | I have a special person who is a real source of comfort to me. |
| 6 | My friends really try to help me. |
| 7 | I can count on my friends when things go wrong. |
| 8 | I can talk about my problems with my family. |
| 9 | I have friends with whom I can share my joys and sorrows. |
| 10 | There is a special person in my life that cares about my feelings. |
| 11 | My family is willing to help me make decisions. |
| 12 | I can talk about my problems with my friends. |

Note. The Significant Other subscale is composed by items 1, 2, 5 and 10. The Family subscale is composed by items 3, 4, 8 and 11. The Friends subscale is composed by items 6, 7, 9 and 12.

Supplementary Table 2. Model parameters of the one-factor model.

| *Factor Loadings* | Posterior mean | 95% CrI | PSRF |
| --- | --- | --- | --- |
| There is a special person who is around when I am in need. | 0.864 | [0.854, 0.874] | - |
| There is a special person with whom I can share joys and sorrows. | 0.882 | [0.872, 0.891] | 1.000 |
| I have a special person who is a real source of comfort to me. | 0.879 | [0.869, 0.887] | 1.001 |
| There is a special person in my life that cares about my feelings. | 0.841 | [0.828, 0.851] | 1.003 |
| My family really tries to help me. | 0.643 | [0.623, 0.664] | 1.002 |
| I get the emotional help and support I need from my family. | 0.711 | [0.692, 0.727] | 1.003 |
| I can talk about my problems with my family. | 0.672 | [0.651, 0.690] | 1.004 |
| My family is willing to help me make decisions. | 0.657 | [0.640, 0.676] | 1.000 |
| My friends really try to help me. | 0.565 | [0.542, 0.588] | 1.001 |
| I can count on my friends when things go wrong. | 0.570 | [0.546, 0.592] | 1.000 |
| I have friends with whom I can share my joys and sorrows. | 0.579 | [0.560, 0.602] | 1.000 |
| I can talk about my problems with my friends. | 0.523 | [0.498, 0.547] | 1.000 |
|  |  |  |  |
| *Intercepts* |  |  |  |
| There is a special person who is around when I am in need. | 4.005 | [3.958, 4.149] | 1.001 |
| There is a special person with whom I can share joys and sorrows. | 4.274 | [4.177, 4.379] | 1.001 |
| I have a special person who is a real source of comfort to me. | 3.978 | [3.891, 4.072] | 1.001 |
| There is a special person in my life that cares about my feelings. | 4.126 | [4.032, 4.227] | 1.002 |
| My family really tries to help me. | 4.291 | [4.192, 4.393] | 1.001 |
| I get the emotional help and support I need from my family. | 4.036 | [3.946, 4.135] | 1.001 |
| I can talk about my problems with my family. | 3.883 | [3.797, 3.979] | 1.001 |
| My family is willing to help me make decisions. | 4.073 | [3.970, 4.160] | 1.001 |
| My friends really try to help me. | 4.543 | [4.443, 4.648] | 1.001 |
| I can count on my friends when things go wrong. | 4.431 | [4.333, 4.535] | 1.000 |
| I have friends with whom I can share my joys and sorrows. | 4.537 | [4.427, 4.643] | 1.000 |
| I can talk about my problems with my friends. | 4.249 | [4.151, 4.347] | 1.001 |
|  |  |  |  |
| *Residual variances* |  |  |  |
| There is a special person who is around when I am in need. | 0.253 | [0.237, 0.271] | 1.001 |
| There is a special person with whom I can share joys and sorrows. | 0.222 | [0.205, 0.239] | 1.002 |
| I have a special person who is a real source of comfort to me. | 0.228 | [0.213, 0.244] | 0.999 |
| There is a special person in my life that cares about my feelings. | 0.293 | [0.275, 0.313] | 0.999 |
| My family really tries to help me. | 0.586 | [0.559, 0.612] | 1.001 |
| I get the emotional help and support I need from my family. | 0.495 | [0.471, 0.521] | 1.000 |
| I can talk about my problems with my family. | 0.549 | [0.524, 0.576] | 0.999 |
| My family is willing to help me make decisions. | 0.568 | [0.543, 0.594] | 1.001 |
| My friends really try to help me. | 0.680 | [0.654, 0.707] | 0.999 |
| I can count on my friends when things go wrong. | 0.675 | [0.649, 0.702] | 1.001 |
| I have friends with whom I can share my joys and sorrows. | 0.664 | [0.637, 0.689] | 1.001 |
| I can talk about my problems with my friends. | 0.726 | [0.701, 0.752] | 1.000 |
| *Social support* | 1.000 | [1.000, 1.000] | 1.002 |
|  |  |  |  |

Note. CrI = credible interval; PSRF = potential scale reduction factor. There is no PSRFs for Item 1 since unstandardized factor loadings were constrained to one during estimation (unit loading identification).

Supplementary Table 3. Model parameters of the two-factor model (SO and FR combined).

| *Factor Loadings* | Posterior mean | 95% CrI | PSRF |
| --- | --- | --- | --- |
| *Friends* |  |  |  |
| There is a special person who is around when I am in need. | 0.890 | [0.882, 0.898] | - |
| There is a special person with whom I can share joys and sorrows. | 0.921 | [0.915, 0.927] | 1.000 |
| I have a special person who is a real source of comfort to me. | 0.906 | [0.899, 0.913] | 1.000 |
| There is a special person in my life that cares about my feelings. | 0.868 | [0.860, 0.877] | 1.000 |
| My friends really try to help me. | 0.513 | [0.488, 0.537] | 0.999 |
| I can count on my friends when things go wrong. | 0.517 | [0.493, 0.542] | 0.999 |
| I have friends with whom I can share my joys and sorrows. | 0.535 | [0.511, 0.558] | 1.000 |
| I can talk about my problems with my friends. | 0.473 | [0.448, 0.499] | 1.000 |
|  |  |  |  |
| *Family* |  |  |  |
| My family really tries to help me. | 0.867 | [0.858, 0.876] | - |
| I get the emotional help and support I need from my family. | 0.927 | [0.920, 0.934] | 0.999 |
| I can talk about my problems with my family. | 0.818 | [0.806, 0.830] | 0.999 |
| My family is willing to help me make decisions. | 0.826 | [0.813, 0.837] | 0.999 |
|  |  |  |  |
| *Intercepts* |  |  |  |
| There is a special person who is around when I am in need. | 4.049 | [3.960, 4.145] | 1.000 |
| There is a special person with whom I can share joys and sorrows. | 4.273 | [4.171, 4.073] | 1.000 |
| I have a special person who is a real source of comfort to me. | 3.980 | [3.883, 4.073] | 1.001 |
| There is a special person in my life that cares about my feelings. | 4.128 | [4.035, 4.230] | 1.002 |
| My family really tries to help me. | 4.544 | [4.444, 4.653] | 1.000 |
| I get the emotional help and support I need from my family. | 4.432 | [4.330, 4.529] | 1.000 |
| I can talk about my problems with my family. | 4.540 | [4.426, 4.645] | 1.000 |
| My family is willing to help me make decisions. | 4.250 | [4.152, 4.350] | 1.000 |
| My friends really try to help me. | 4.291 | [4.191, 4.387] | 1.002 |
| I can count on my friends when things go wrong. | 4.037 | [3.951, 4.134] | 1.001 |
| I have friends with whom I can share my joys and sorrows. | 3.884 | [3.795, 3.977] | 1.001 |
| I can talk about my problems with my friends. | 4.072 | [3.986, 4.172] | 1.001 |
|  |  |  |  |
| *Residual variances* |  |  |  |
| There is a special person who is around when I am in need. | 0.207 | [0.193, 0.221] | 0.999 |
| There is a special person with whom I can share joys and sorrows. | 0.151 | [0.140, 0.163] | 0.999 |
| I have a special person who is a real source of comfort to me. | 0.179 | [0.167, 0.192] | 1.000 |
| There is a special person in my life that cares about my feelings. | 0.246 | [0.231, 0.260] | 0.999 |
| My family really tries to help me. | 0.737 | [0.712, 0.762] | 1.000 |
| I get the emotional help and support I need from my family. | 0.733 | [0.707, 0.753] | 1.000 |
| I can talk about my problems with my family. | 0.714 | [0.689, 0.739] | 1.000 |
| My family is willing to help me make decisions. | 0.776 | [0.753, 0.801] | 1.000 |
| My friends really try to help me. | 0.249 | [0.233, 0.264] | 1.000 |
| I can count on my friends when things go wrong. | 0.141 | [0.129, 0.154] | 0.999 |
| I have friends with whom I can share my joys and sorrows. | 0.330 | [0.312, 0.350] | 1.000 |
| I can talk about my problems with my friends. | 0.318 | [0.299, 0.339] | 0.999 |
| *Friends* | 1.000 | [1.000, 1.000] | 1.000 |
| *Family* | 1.000 | [1.000, 1.000] | 0.999 |
|  |  |  |  |
| *Factor correlations* |  |  |  |
| *Friends ~~ Family* | 0.663 | [0.644, 0.683] | 0.999 |

Note. CrI = credible interval; PSRF = potential scale reduction factor. There are no PSRFs for Item 1 and Item 3 since unstandardized factor loadings were constrained to one during estimation (unit loading identification).

Supplementary Table 4. Model parameters of the two-factor model (FA and FR combined).

| *Factor Loadings* | Posterior mean | 95% CrI | PSRF |
| --- | --- | --- | --- |
| *Significant Other* |  |  |  |
| There is a special person who is around when I am in need. | 0.898 | [0.891 - 0.905] | - |
| There is a special person with whom I can share joys and sorrows. | 0.936 | [0.931 - 0.941] | 1.000 |
| I have a special person who is a real source of comfort to me. | 0.907 | [0.901 - 0.915] | 1.000 |
| There is a special person in my life that cares about my feelings. | 0.873 | [0.864 - 0.881] | 1.000 |
|  |  |  |  |
| *Family* |  |  |  |
| My friends really try to help me. | 0.632 | [0.611 - 0.654] | - |
| I can count on my friends when things go wrong. | 0.638 | [0.616 - 0.660] | 1.002 |
| I have friends with whom I can share my joys and sorrows. | 0.620 | [0.597 - 0.643] | 1.001 |
| I can talk about my problems with my friends. | 0.587 | [0.562 - 0.610] | 1.001 |
| My family really tries to help me. | 0.815 | [0.801 - 0.828] | 1.001 |
| I get the emotional help and support I need from my family. | 0.863 | [0.851 - 0.874] | 0.999 |
| I can talk about my problems with my family. | 0.818 | [0.807 - 0.830] | 0.999 |
| My family is willing to help me make decisions. | 0.821 | [0.808 - 0.833] | 0.999 |
|  |  |  |  |
| *Intercepts* |  |  |  |
| There is a special person who is around when I am in need. | 4.550 | [4.449 - 4.654] | 1.000 |
| There is a special person with whom I can share joys and sorrows. | 4.432 | [4.328 - 4.536] | 1.000 |
| I have a special person who is a real source of comfort to me. | 4.537 | [4.432 - 4.647] | 1.001 |
| There is a special person in my life that cares about my feelings. | 4.249 | [4.147 - 4.359] | 1.002 |
| My family really tries to help me. | 4.290 | [4.183 - 4.385] | 1.000 |
| I get the emotional help and support I need from my family. | 4.035 | [3.939 - 4.136] | 1.000 |
| I can talk about my problems with my family. | 3.882 | [3.797 - 3.984] | 1.000 |
| My family is willing to help me make decisions. | 4.071 | [3.975 - 4.171] | 1.000 |
| My friends really try to help me. | 4.048 | [3.953 - 4.147] | 1.002 |
| I can count on my friends when things go wrong. | 4.273 | [4.168 - 4.370] | 1.001 |
| I have friends with whom I can share my joys and sorrows. | 3.979 | [3.887 - 4.074] | 1.001 |
| I can talk about my problems with my friends. | 4.126 | [4.026 - 4.222] | 1.001 |
|  |  |  |  |
| *Residual variances* |  |  |  |
| There is a special person who is around when I am in need. | 0.600 | [0.573 - 0.627] | 1.000 |
| There is a special person with whom I can share joys and sorrows. | 0.593 | [0.565 - 0.620] | 1.000 |
| I have a special person who is a real source of comfort to me. | 0.616 | [0.587 - 0.644] | 1.000 |
| There is a special person in my life that cares about my feelings. | 0.655 | [0.628 - 0.684] | 1.000 |
| My family really tries to help me. | 0.335 | [0.314 - 0.358] | 1.000 |
| I get the emotional help and support I need from my family. | 0.255 | [0.237 - 0.277] | 1.001 |
| I can talk about my problems with my family. | 0.330 | [0.311 - 0.349] | 1.000 |
| My family is willing to help me make decisions. | 0.326 | [0.306 - 0.348] | 1.000 |
| My friends really try to help me. | 0.194 | [0.180 - 0.206] | 0.999 |
| I can count on my friends when things go wrong. | 0.124 | [0.114 - 0.134] | 1.001 |
| I have friends with whom I can share my joys and sorrows. | 0.177 | [0.164 - 0.189] | 1.000 |
| I can talk about my problems with my friends. | 0.238 | [0.224 - 0.254] | 1.000 |
| *Significant Other* | 1.000 | [1.000 - 1.000] | 1.001 |
| *Family* | 1.000 | [1.000 - 1.000] | 1.000 |
|  |  |  |  |
| *Factor correlations* |  |  |  |
| *Significant Other ~~ Family* | 0.672 | [0.651, 0.691] | 1.002 |

Note. CrI = credible interval; PSRF = potential scale reduction factor. There are no PSRFs for Item 1 and Item 6 since unstandardized factor loadings were constrained to one during estimation (unit loading identification).

Supplementary Table 5. Model parameters of the two-factor model (FA and SO combined).

| *Factor Loadings* | Posterior mean | 95% CrI | PSRF |
| --- | --- | --- | --- |
| *Significant Other* |  |  |  |
| There is a special person who is around when I am in need. | 0.888 | [0.880 - 0.896] | - |
| There is a special person with whom I can share joys and sorrows. | 0.913 | [0.907 - 0.919] | 1.000 |
| I have a special person who is a real source of comfort to me. | 0.901 | [0.893 - 0.908] | 1.000 |
| There is a special person in my life that cares about my feelings. | 0.864 | [0.855 - 0.874] | 1.000 |
| My family really tries to help me. | 0.605 | [0.584 - 0.627] | 1.000 |
| I get the emotional help and support I need from my family. | 0.674 | [0.654 - 0.692] | 1.000 |
| I can talk about my problems with my family. | 0.630 | [0.610 - 0.652] | 1.000 |
| My family is willing to help me make decisions. | 0.614 | [0.593 - 0.634] | 1.000 |
| *Friends* |  |  |  |
| My friends really try to help me. | 0.860 | [0.850 - 0.871] | - |
| I can count on my friends when things go wrong. | 0.882 | [0.871 - 0.890] | 1.001 |
| I have friends with whom I can share my joys and sorrows. | 0.883 | [0.873 - 0.892] | 1.003 |
| I can talk about my problems with my friends. | 0.843 | [0.832 - 0.854] | 1.003 |
|  |  |  |  |
| *Intercepts* |  |  |  |
| There is a special person who is around when I am in need. | 4.048 | [3.956 - 4.142] | 1.000 |
| There is a special person with whom I can share joys and sorrows. | 4.271 | [4.179 - 4.381] | 1.001 |
| I have a special person who is a real source of comfort to me. | 3.979 | [3.888 - 4.076] | 1.000 |
| There is a special person in my life that cares about my feelings. | 4.126 | [4.029 - 4.221] | 1.000 |
| My family really tries to help me. | 4.290 | [4.194 - 4.393] | 1.001 |
| I get the emotional help and support I need from my family. | 4.036 | [3.944 - 4.133] | 1.000 |
| I can talk about my problems with my family. | 3.883 | [3.794 - 3.983] | 1.000 |
| My family is willing to help me make decisions. | 4.074 | [3.978 - 4.164] | 1.000 |
| My friends really try to help me. | 4.546 | [4.447 - 4.657] | 1.000 |
| I can count on my friends when things go wrong. | 4.431 | [4.327 - 4.531] | 1.000 |
| I have friends with whom I can share my joys and sorrows. | 4.538 | [4.431 - 4.643] | 1.000 |
| I can talk about my problems with my friends. | 4.249 | [4.147 - 4.347] | 1.001 |
|  |  |  |  |
| *Residual variances* |  |  |  |
| There is a special person who is around when I am in need. | 0.212 | [0.197 - 0.226] | 1.000 |
| There is a special person with whom I can share joys and sorrows. | 0.166 | [0.155 - 0.178] | 1.000 |
| I have a special person who is a real source of comfort to me. | 0.189 | [0.176 - 0.203] | 1.000 |
| There is a special person in my life that cares about my feelings. | 0.253 | [0.237 - 0.269] | 0.999 |
| My family really tries to help me. | 0.634 | [0.607 - 0.659] | 1.000 |
| I get the emotional help and support I need from my family. | 0.545 | [0.521 - 0.572] | 1.000 |
| I can talk about my problems with my family. | 0.603 | [0.577 - 0.630] | 1.000 |
| My family is willing to help me make decisions. | 0.623 | [0.599 - 0.649] | 0.999 |
| My friends really try to help me. | 0.261 | [0.242 - 0.277] | 1.000 |
| I can count on my friends when things go wrong. | 0.222 | [0.207 - 0.241] | 1.000 |
| I have friends with whom I can share my joys and sorrows. | 0.220 | [0.204 - 0.237] | 1.001 |
| I can talk about my problems with my friends. | 0.290 | [0.271 - 0.308] | 1.000 |
| *Significant Other* | 1.000 | [1.000, 1.000] | 1.001 |
| *Friends* | 1.000 | [1.000, 1.000] | 1.000 |
|  |  |  |  |
| *Factor correlations* |  |  |  |
| *Significant Other ~~ Friends* | 0.548 | [0.522, 0.572] | 1.000 |

Note. CrI = credible interval; PSRF = potential scale reduction factor. There are no PSRFs for Item 1 and Item 6 since unstandardized factor loadings were constrained to one during estimation (unit loading identification).

Supplementary Table 6. Fit statistics for the factor analytical models.

|  | χ^2^ | *p* | *df* | *p-*value | RMSEA | 90% CI | CFI | 90% CI^a^ |
| --- | --- | --- | --- | --- | --- | --- | --- | --- |
| ML |  |  |  |  |  |  |  |  |
| *One-factor structure* | 17344.35 | 36.00 | 54.00 | <0.001 | 0.288 | [0.284, 0.291] | 0.596 | - |
| *Two-factor structure*  *(FR and SO)* | 10867.74 | 37.00 | 53.00 | <0.001 | 0.230 | [0.226, 0.233] | 0.747 | - |
| *Two-factor structure*  *(FA and FR)* | 10089.52 | 37.00 | 53.00 | <0.001 | 0.221 | [0.218, 0.225] | 0.765 | - |
| *Two-factor structure*  *(FA and SO)* | 8886.30 | 39.70 | 53.00 | <0.001 | 0.208 | [0.204, 0.211] | 0.793 | - |
| *Three-factor structure*  *(SO, FA and FR)* | 1609.80 | 39.00 | 51.00 | <0.001 | 0.089 | [0.085, 0.093] | 0.964 | - |

Note. CFA = confirmatory factor analysis; χ2 = chi-square; *p* = number of parameters; df = degrees of freedom; RMSEA = root mean square error of approximation; CFI = comparative fit index; ML = maximum likelihood. a. Since the CFI has an unknown sampling distribution, 95% CIs could not be calculated.

**
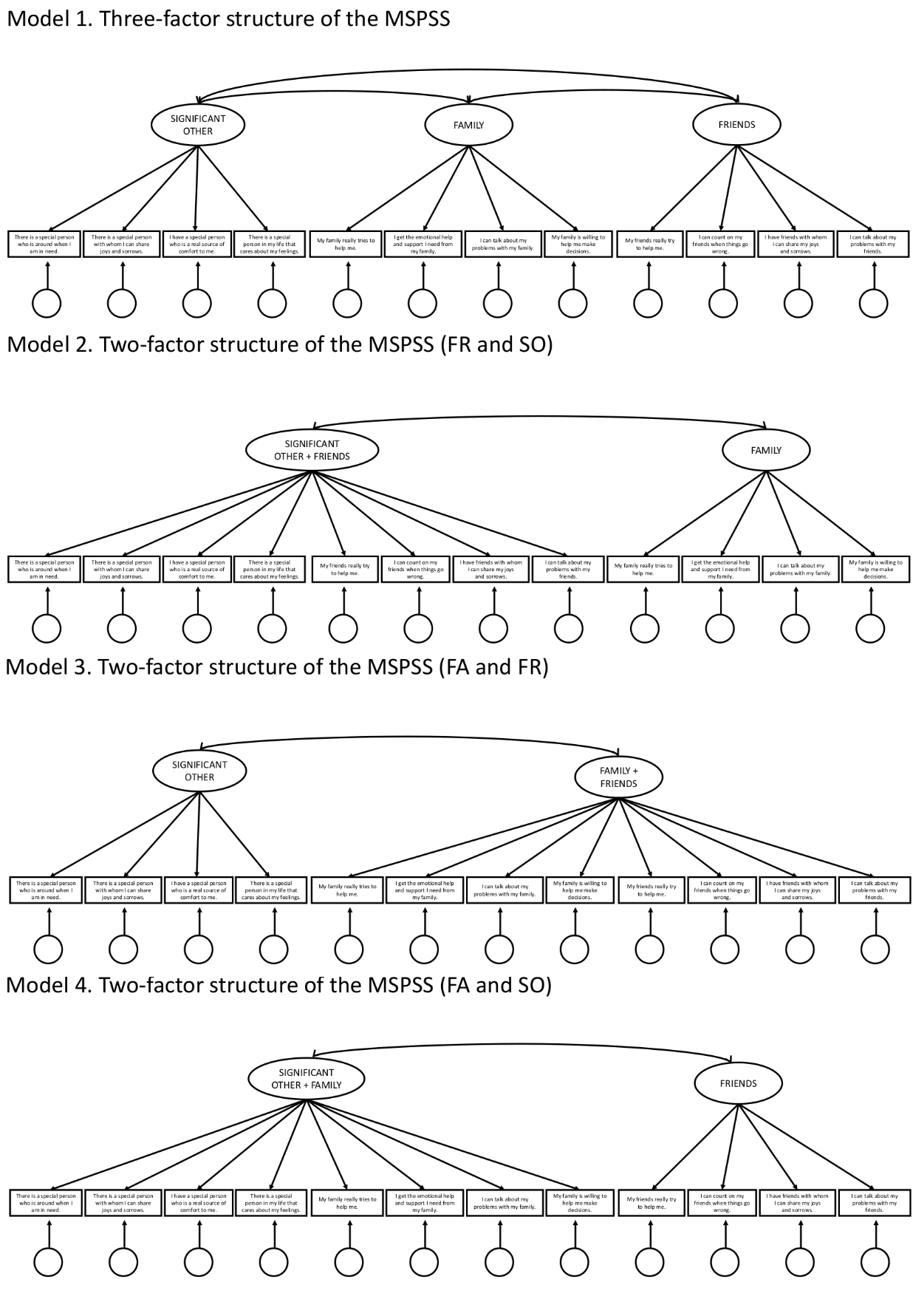
**

**Supplementary Figure 1.** Factorial structures of the MSPSS.

**
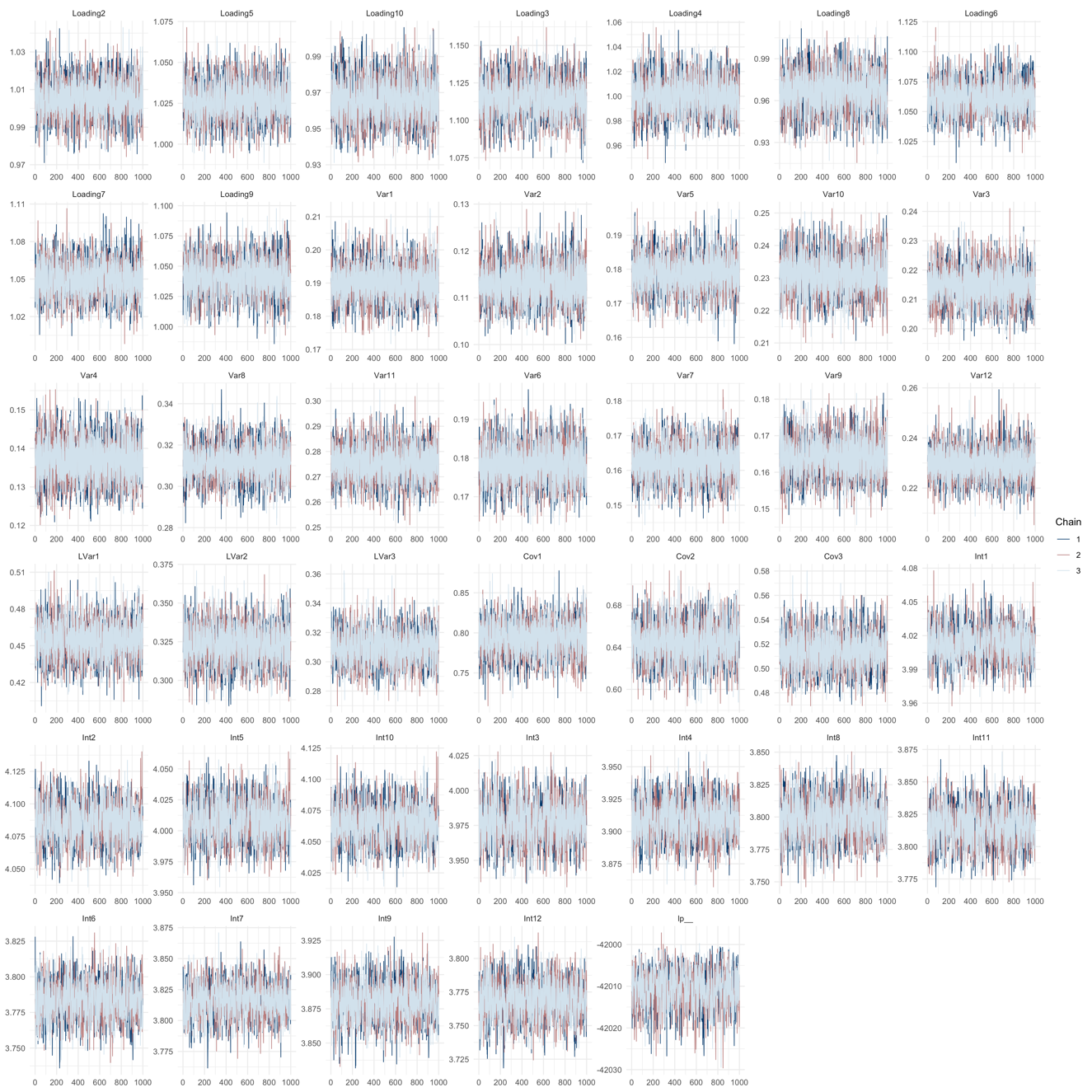
**

**Supplementary Figure 2.** Trace plots of the three-factor model (Significant Other, Family and Friends) parameters. Note. Numbers = item number; Loading = factor loading; Var = residual variance; Lvar = latent variables’ variance; Cov = latent variables’ covariance; Int = intercept. The x-axis indicates the number of Markov chains. The y-axis indicates the parameter value. Red traces represent the first Markov chain, blue traces represent the second Markov chain and purple traces represent the third Markov chain.
